# Around the Hospitals

**Published:** 1893-06-10

**Authors:** 


### Around the Hospitals.

Notice to the Managers of Institutions.—Special space is now reserved for the insertion of notices of hospital meetings and festivals. The Editor requests that all notices may reach The Hospital Office, 428, Strand, at least one week before the date of each meeting.

**The Royal Berkshire Hospital.**—The report of this institution for the present year commences by the remark that it contains no feature of special interest. Nevertheless, the record is a very satisfactory one. In-patients have numbered 1,247, and out-patients amounted to 2,001. Subscriptions show a steady increase, substantial egacies were received, and the Hospital Saturday Collection was the largest yet made, the great increase by 2,684 in the actual number of coins testifying to added general interest in the institution. Additions and improvements have been made during the past year, although not of a very important character.

**The London Lock Hospital**, which supplies a very real and pressing want as a rescue home also, has, we are glad to learn, been well recognised during the year, and contributors will be encouraged to learn that the expenses have been so carefully supervised that a reduction of £500 was made on the expenditure of previous years. The female hospital in the Harrow Road has a ladies' committee, which pays every attention to the comfort and efficiency of the management of the institution. Testimony of the good work done, both medically and morally, within the hospital is frequently received. The objects of the institution include the placing of the inmates in respectable positions on leaving, and useful instruction is given to the female patients during the year's residence required. The hospital is most worthy of support, and Mr. Grant, the energetic secretary, is to be congratulated on his successful manner of arousing public sympathy on its behalf.

**The Norfolk and Norwich Hospital.**—The report of this hospital, read at the annual meeting, is a somewhat bewildering one. We are told first that the increased average cost per patient for the year is due to the fact that the diet has been remodelled on a more liberal scale, an extra meal being added during the day, as previously the patients received no food from half-past five in the evening until half-past seven the next morning. A statement that a decrease of £300 was shown on articles of consumption was received by the meeting with applause. This decrease is probably due to the diminished number of patients treated during the year, a manner of lessening expenses hardly to be a cause for congratulation. The decrease of the gas bill by £57 during the quarter is unquestionably an improvement, though it points to great previous extravagance to permit such a very large diminution. Improvements in the nursing departments have been effected. One point in the history of the Norfolk and Norwich Hospital is much to be regretted, namely, that every year since its foundation ten years ago a falling off of £100 per annum in subscriptions is noticeable up to 1892. During his year of office as Mayor, Mr. G. Chamberlain instituted a fund which greatly benefited the hospital. The decrease in subscriptions during the same year, 1892, was far less marked, and so it is to be hoped that a better future is in store for the institution.

